# Oxygen uptake during functional activities after stroke—Reliability and validity of a portable ergospirometry system

**DOI:** 10.1371/journal.pone.0186894

**Published:** 2017-10-24

**Authors:** Tor Ivar Gjellesvik, Berit Brurok, Arnt Erik Tjønna, Tom Tørhaug, Torunn Askim

**Affiliations:** 1 Department of Neuromedicine and Movement Science, Faculty of Medicine and Health Science, NTNU, Norwegian University of Science and Technology, Trondheim, Norway; 2 Department of Physical Medicine and Rehabilitation, St. Olav’s University Hospital, Trondheim, Norway; 3 Centre for Elite Sports Research, Department of Neuromedicine and Movement Science, NTNU, Norwegian University of Science and Technology, Trondheim, Norway; 4 Department of Circulation and Medical Imaging, Faculty of Medicine and Health Science, NTNU, Norwegian University of Science and Technology, Trondheim, Norway; 5 NeXt Move, NTNU, Trondheim, Norway; Sao Paulo State University, BRAZIL

## Abstract

**Background:**

People with stroke have a low peak aerobic capacity and experience increased effort during performance of daily activities. The purpose of this study was to examine test-retest reliability of a portable ergospirometry system in people with stroke during performance of functional activities in a field-test. Secondary aims were to examine the proportion of oxygen consumed during the field-test in relation to the peak-test and to analyse the correlation between the oxygen uptake during the field-test and peak-test in order to support the validity of the field-test.

**Methods:**

With simultaneous measurement of oxygen consumption, participants performed a standardized field-test consisting of five activities; walking over ground, stair walking, stepping over obstacles, walking slalom between cones and from a standing position lifting objects from one height to another. All activities were performed in self-selected speed. Prior to the field-test, a peak aerobic capacity test was performed. The field-test was repeated minimum 2 and maximum 14 days between the tests. ICC_2,1_ and Bland Altman tests (Limits of Agreement, LoA) were used to analyse test-retest reliability.

**Results:**

In total 31 participants (39% women, mean (SD) age 54.5 (12.7) years and 21.1 (14.3) months’ post-stroke) were included. The ICC_2,1_ was ≥ 0.80 for absolute V̇O_2_, relative V̇O_2_, minute ventilation, CO_2_, respiratory exchange ratio, heart rate and Borgs rating of perceived exertion. ICC_2,1_ for total time to complete the field-test was 0.99. Mean difference in steady state V̇O_2_ during Test 1 and Test 2 was -0.40 (2.12) The LoAs were -3.75 and 4.51. Participants spent 60.7% of their V̇O_2_peak performing functional activities. Correlation between field-test and peak-test was 0.689, p = 0.001 for absolute and 0.733, p = 0.001 for relative V̇O_2_.

**Conclusions:**

This study presents first evidence on reliability of oxygen uptake during performance of functional activities after stroke, showing very good test-retest reliability. The secondary analysis showed that the amount of energy spent during the field-test relative to the peak-test was high and the correlation between the two test was good, supporting the validity of this method.

## Introduction

Physical activity and exercise is highly recommended in the chronic phase after stroke to sustain functions gained in rehabilitation and as part of long-term secondary prevention of recurrent vascular events. [1–3] However, studies have found that stroke survivors have a reduced peak aerobic capacity in addition to an elevated energy expenditure (ie. oxygen uptake) in walking. [4] During performance of daily activities (ADL), oxygen uptake (V̇O_2_) increases in line with the demand of the activity. [5] After stroke, the V̇O_2_ in walking activities has been found to be nearly twice as high as in age, sex and weight matched healthy persons. [6, 7]

One limitation from research assessing V̇O_2_ during walking in the stroke population, is the lack of knowledge of their V̇O_2_peak, making it impossible to calculate the relative strain during ADL, [8–11] which has been shown to be high in patients with other cardiovascular diseases. [12, 13]

Test-retest reliability of an incremental V̇O_2_peak has shown to be good in chronic stroke survivors. [14] Reliable and valid methods are also needed to examine V̇O_2_ and energy expenditure during ADL in the stroke population. Different portable metabolic ergospirometry systems have been developed to meet this demand. [15] However, the measurement properties of these systems have been poorly investigated in the chronic stroke population. So far, only two studies have assessed the test-retest reliability of portable devices, showing good to excellent reliability during treadmill walking and over ground walking in people with chronic stroke. [8] [10] The MetaMax system (Cortex Biophysik, Leipzig, Germany) has been validated against the Douglas-Bag technique which is known to be the gold-standard in ergospirometry testing and has been found to be valid in healthy individuals. [16] Hence, assessing the concurrent validity against V̇O2peak and the relative strain during ADL would be of great interest to support the validity of this method in the stroke population.

In order to evaluate the reliability of V̇O_2_ during submaximal functional activities we designed a standardized field-test to measure V̇O_2_, related cardiopulmonary variables, heart rate (HR) and rating of perceived exertion (RPE) on the Borg scale during the performance of five functional activities in a sample of stroke participants. We also wanted to determine the proportion of energy expenditure (i.e. V̇O_2_) during the field-test in relation to the peak-test people after stroke specifically were consuming during the performance of the functional activities. Furthermore, to support the validity of this system we wanted to assess the correlation between V̇O_2_ during the field-test and peak aerobic capacity (V̇O_2_peak).

The specific aims were: I) To determine test-retest reliability of the MetaMax II portable ergospirometry system for measuring V̇O_2_ and related variables during five functional activities in people with stroke. II) To assess the proportion of V̇O_2_ consumed during self-paced functional activities in relation to V̇O_2_peak. III) To assess the correlation between the V̇O_2_ consumed during self-paced functional activities and during the peak-test.

Our primary hypothesis was that test-retest reliability of cardiopulmonary ergospirometry measured by a portable system during five functional activities was good with an ICC ≥ 0.80.

Our secondary hypotheses were that people after stroke spend more than 50% of their V̇O_2_peak during performance of functional activities in a standardized field-test and that the correlation between the V̇O_2_peak and the V̇O_2_ consumed during functional tasks was strong (r>0.60).

## Methods

### Participants

Participants were recruited from the Stroke Unit and a from a rehabilitation centre at Trondheim University Hospital, Norway. Inclusion started in May 2014 and ended in May 2015. Groups of 4–5 participants were included at a time. Inclusion criteria were; diagnosis of stroke, ≥ 4 months and ≤ 5 years from onset, 18 to 75 years old and living in near proximity to test sites. In addition, a minimum score on the modified Rankin Scale (mRS) of 0–3 was required. Participants were excluded if they had serious heart or lung diseases preventing them from being tested or a resting blood pressure >180/100. Participants were examined by a medical doctor before final inclusion to the study. All participants received oral and written information about the study and gave written consent. The study was approved by the Regional Committee for Medical and Health Research Ethics (REK-Vest, 2014/55).

### Study design

**Fig 1:**
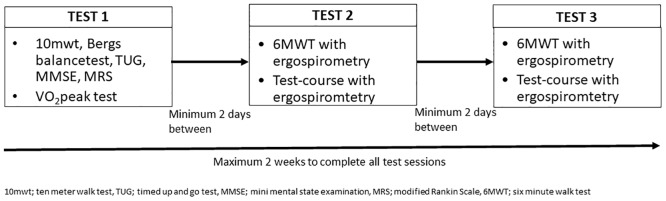
Study design.

This study had a test-retest design with an initial baseline assessment of V̇O_2_peak and functional tests at inclusion. Two to fourteen days later the test and retest of the ergospirometry system during functional activities in a standardized field-test was performed, with a minimum of two days between the tests. Fig 1 depicts details of the study design. No clinical change in V̇O_2_peak was expected during this period as the participants were asked to maintain their daily activity levels and none of the participants took part in rehabilitation or any intensive training programme.

### Procedures

The same MetaMax II ergospirometry system and software was used during all test sessions. Before the V̇O_2_peak treadmill- test and the field-test, the oxygen (O_2_) and carbon dioxide (CO_2_) sensors were calibrated using a gas mixture sample of 15.0% O_2_ and 5.0% CO_2_. In addition, room air calibration and pressure were performed before each test. The turbine volume transducer was calibrated using a 3.0-L syringe (Hans Rudolph Inc., Kansas, USA). A facemask was positioned over the participant’s mouth and nose with the volume transducer attached. V̇O_2_ and related variables were measured continuously and averaged over 10-s periods. During field-testing, each participant wore the mixing chamber, transmitter and battery in a harness attached to their back in a vest (total of 1.25 kg). Cardiopulmonary variables were sent from the transmitter to a receiver and real-time measures were displayed with the MetaSoft 1 program on a laptop. The equipment has been validated for ventilatory and metabolic demand previously. [17] Measured cardiopulmonary performance parameters were V̇O_2_ for both absolute (L/min) and relative to body mass (mL/kg/min), carbon dioxide output (VCO_2_), minute ventilation rate (V_E_) and respiratory exchange ratio (RER). In addition, heart rate (HR) was recorded with Polar heart rate monitor (Polar Electro, Finland), and participants level of perceived exertion on the Borg scale were registered. [18] Immediately after the treadmill peak aerobic test we analysed blood lactate level drawn from the fingertip with the Lactate Pro^™^ Analyzer (Arkray KDK, Japan).

### Test of balance, gait and cognitive function

In order to retrieve relevant background information on the participant’s physical and cognitive function, a set of functional tests were performed at inclusion (Test 1). Bergs balance test was applied to assess balance, [19] 10 meter walk test was applied to assess normal and maximal gait speed, [20] The Scandinavian Stroke Scale[21] was applied to assess severity of stroke, the modified Rankin Scale[22] was applied to assess independence in daily living and the Mini Mental State Examination test (MMSE) [23] was applied to assess cognitive function.

### V̇O_2_peak-test

The medical doctor evaluated individual electronic patient records and performed a preparticipation health risk screening according to the guidelines for exercise testing and prescription, established by the American College of Sports Medicine (ACSM)[24]. Their body mass and height was measured and rounded to the nearest 0.5 cm. The test procedure was explained to each participant and they were familiarized to treadmill walking and the test equipment. An individualized ramped protocol was used to assess each participant’s V̇O_2_peak. After a 10–15 minutes’ warm-up period, their maximal walking speed was assessed and the test started with gradually increasing inclination by 2% every minute to voluntational fatigue. Verbal encouragements were given during the test and one study assistant took care of the participants’ safety. The V̇O_2_peak–test was conducted in a laboratory setting. Borgs RPE, peak HR and blood lactate levels were registered at termination of the test. Test termination criteria to assess V̇O_2_peak were a levelling off in V̇O_2_ despite further increase in work load, RER ≥1.05, blood lactate concentration ≥ 7.0 mmol/L and RPE ≥15. For the data analysis, the average of the 3 highest continuous 10-second V̇O2-measures was used in the calculation of V̇O_2_peak.

### V̇O_2_ during the Six-minute walk test (6MWT)

Before the field-test, each participant performed a 6MWT with simultaneous measurements of V̇O_2_ and HR recordings wearing the portable MetaMax II device and a HR monitor. Participants were instructed to walk as far as possible during the 6 minutes between two cones 25 meter apart in a hospital corridor. They were instructed to walk at their safest but fastest walking speed. Information on remaining time was given every minute. No other encouragements were given during the test. HR was registered and averaged over the last 30 seconds of the test and the total distance in meters were registered. RPE was registered immediately after termination of the test.

### V̇O_2_ during the standardized field-test

**Fig 2:**
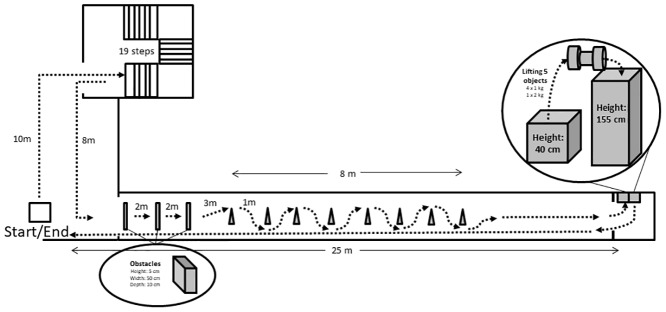
Schematic illustration of the field-test.

The field-test consisted of 5 different functional activities; walking over ground, walking with changing directions, walking over obstacles, stair walking (1 floor—19 steps) and from a standing position, lifting 5 (4x1 and 1x2 kg) objects from one height to another and back again. The starting positions of the objects were 55 cm above floor level and they were to be lifted to a height of 155 cm with their non-affected arm. Fig 2 shows an illustration of the field-test. On both test days, under supervision and instructions from the study coordinator, the participants performed a familiarization round in the field-test without wearing the portable metabolic ergospirometry equipment. The actual test started with the participant sitting for 3 minutes on a chair wearing the portable equipment attached to reach steady state and get used to the equipment. Each participant performed two laps in the field-test without a break between. Time used to complete the test was registered with a stopwatch.

For field-testing, participants were instructed to wear the same shoes/clothing and if applicable orthoses and assistive devices for the two test days. In contrast to the 6MWT, the participants were instructed to walk at “their comfortable” speed during the field-test. In detail, they were told to walk and perform the activities, as they would do in their own home. The average of the last 30 seconds (3 continuous measurements) for all cardiopulmonary variables and HR were used in the calculations. At the end of the field-test, participants were asked to report their level of perceived exertion on the Borg 6–20 scale.

Any adverse events during or after the tests were recorded.

The same testers performed the test and the re-tests. However, the results from the first test was not available for the testers when the re-test was performed.

### Statistical analysis

Descriptive statistics are presented as means and standard deviations (SD). The test-retest reliability was calculated using a 2-way mixed intra class correlation coefficient (ICC_2,1_). The accuracy of the ICC_2,1_ was quantified using the 95% confidence interval (CI). The Pearson correlation coefficient was used to analyze the correlation between V̇O_2_ during the peak-test and the field-test. To test the limits of agreement in V̇O_2_ (mL/kg/min) between Test 1 and Test 2 a Bland Altman analysis was performed and a Bland Altman plot was used to depict the difference in steady state VO_2_ against the mean of the two measurements. Horizontal lines were drawn at the mean difference and the limits of agreement (LoA) were defined as the mean difference plus or minus 1.96 times the SD of the difference between Test 1 and Test 2. All statistical analyzes were performed in SPSS Inc. Version 22 (Chicago, Ill., USA) with an alpha level >0.05.

## Results

In total 31 participants (12 women) were included in the study. Participant’s characteristics are depicted in Table 1. The mean (SD) age was 54.5 (12.7) years, the normal gait speed was 0.94 (0.52) m/s.

**Table 1:** Participant’s characteristics at inclusion (n = 31).

Characteristics	Mean (±SD)
Age (yrs)	54.5 (12.7)
Time since stroke (months)	21.1 (14.3)
Weight (kg)	80.3 (14.7)
Height (cm)	175.8 (10.0)
BMI	26.3 (14.3)
MRS (0–6)	1.7 (0.7)
SSS (0–58)	53.0 (5.0)
MMSE (0–30)	27.8 (2.5)
Bergs balance test (0–56)	52.9 (4.4)
10 meter walk test normal (m/s)	0.94 (0.52)
10 meter walk test maximal (m/s)	0.69 (0.48)
Assistive devices (n)	9
Crutch	8
	9
AFO	8

SD, standard deviation; BMI, body mass index; MRS, modified Rankin scale; SSS, Scandinavian Stroke Scale; MMSE, Mini Mental State Examination

**Fig 3:**
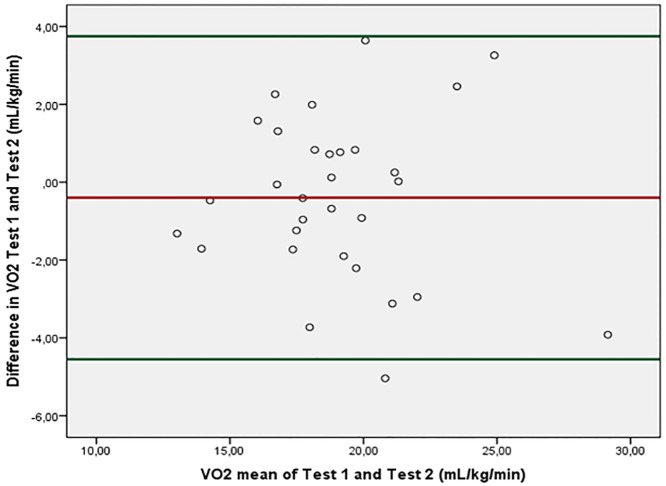
Bland-Altman plot of within subject change in VO_2_ between Test 1 and Test 2 (mL/kg/min). Red horizontal line represents the mean difference between tests. Green horizontal lines are reference lines representing 5% and 95% confidence intervals (± 1.96 SD).

For all cardiopulmonary variables except RER the ICC between Test 1 and Test 2 was ≥ .80. Moreover, Bland Altman plots of within subject change in steady state V̇O_2_ as a function of mean steady state V̇O_2_ during Test 1 and Test 2 (Fig 3) demonstrate a mean difference of -0.40 (2.12) The upper LoA was drawn at 4.51, while the lower LoA was drawn at -3.75. For V̇O_2_, both absolute values and related to body mass, the participants consumed more than 60% of their total aerobic capacity performing self-paced functional activities. V̇O_2_ during the 6MWT showed similar results (Tables 2 and 3).

**Table 2:** Reliability measures for the 6MWT (n = 31).

Variable	Test 1	Test 2	ICC_2,1_	95% CI
Distance (m)	500.2 (163.6)	506.9 (164.1)	0.99	0.99–0.99
V̇O_2_ (mL/kg/min)	19.9 (5.2)	20.7 (5.75)	0.90	0.89–0.97
V̇O_2_ (L/min)	1.6 (0.47)	1.65 (0.49)	0.93	0.86–0.98
CO_2_ (L/min)	1.4 (0.47)	1.48 (0.44)	0.82	0.66–0.91
VE (L/min)	43.3 (13.22)	47.2 (13.97)	0.77	0.57–0.88
RER	0.89 (0.05)	0.91 (0.05)	0.56	0.27–0.76
HR	126 (20.3)	124.1 (21.18)	0.94	0.89–0.97
RPE	12.7 (2.1)	12.2 (2.68)	0.82	0.66–0.91
Lactate (mmol/L)	7.58 (2.23)	-	-	-

CI, confidence interval; V̇O_2_, oxygen uptake; CO_2_, carbon dioxide output; VE, minute ventilation; RER, respiratory exchange ratio; HR, heart rate; RPE, rating of perceived exertion

**Table 3:** Reliability measures for the field-test (n = 31).

	Test 1	Test 2	ICC_2,1_	95% CI
Time (min)	4.05 (2.50)	4.02 (2.57)	0.99	0.98–0.99
V̇O_2_ (mL/kg/min)	18.84 (3.28)	19.23 (3,45)	0.80	0.62–0.90
V̇O_2_ (L/min)	1.51 (0.35)	1.53 (0.36)	0.88	0.78–0.94
CO_2_ (L/min)	1.28 (0.29)	1.31 (0.32)	0.80	0.63–0.89
VE (L/min)	40.25 (10.68)	42.12 (13.09)	0.82	0.66–0.91
RER	0.85 (0.04)	0.86 (0.04)	0.53	0.23–0.75
HR	121.3 (19.12)	120.65 (19.86)	0.91	0.63–0.90
RPE	12.0 (2.13)	12.1 (2.77)	0.81	0.64–0.90

CI, confidence interval; V̇O_2_, oxygen uptake; CO_2_, carbon dioxide output; VE, minute ventilation; RER, respiratory exchange ratio; HR, heart rate; RPE, rating of perceived exertion

One person did not complete the V̇O_2_peak test because of comorbidity influencing the well-being of the person at the time of the V̇O_2_peak-test. However, the field-test was performed as planned. The mean (SD) V̇O_2_ was 33.59 (9.54) mL/kg/min during the peak-test and 19.0 (3.2) mL/kg/min during the field-test (Table 4). The time used to complete the field-test ranged from less than 3 minutes to more than 12 minutes (Table 4).

**Table 4:** Relevant variables at V̇O_2_peak, during field-testing and proportion of aerobic capacity (n = 30).

	V̇O_2_peak-test	V̇O_2_ in Field-test	% of VO_2_peak	VO_2_ in 6MWT	% of VO_2_peak	Pearson’s r*
Variables	Mean (SD)	Mean (SD)	Mean (SD)	Mean (SD)	Mean (SD)	
L/min	2.7 (0.9)	1.52 (0.4)	60.7 (12.0)	1.6 (0.5)	62.0 (12.7)	0.689**
mL/kg/min	33.6 (9.5)	19.0 (3.2)	60.7 (11.1)	20.3 (5.4)	62.2 (12.6)	0.733**
CO_2_ (L/min)	2.8 (1.0)	1.3 (0.3)	50.7 (10.9)	1.5 (0.5)	54.5 (12.8)	0.760**
RER	1.05 (0.06)	0.86 (0.03)	82.3 (5.7)	0.9 (0.5)	85.6 (4.9)	0.143
VE (L/min)	88.3 (29.1)	41.2 (11.4)	50.0 (13.7)	45.2 (12.8)	54.8 (18.9)	0.477
HR	167.8 (18.5)	121 (19.1)	73.4 (11.6)	125.1(20.5)	74.7 (10.9)	0.320
RPE	16.0 (1.5)	12.0 (2.4)	75.5 (16.4)	12.4 (2.3)	76.2 (15.1)	-0.179

SD: standard deviation; CO_2_: carbon dioxide output; RER: respiratory exchange ratio; VE: minute ventilation; HR: heart rate; RPE: rating of perceived exertion *Correlation betweenV̇O_2_peak-test and V̇O_2_ in the field-test,

**p<0.001

A moderate correlation was found for both absolute (L/min) and relative (mL/kg/min) V̇O_2_ values between the treadmill peak-test and the field-test, r = 0.689 and r = 0.733, p>0.001, respectively (Table 4).

The rating on the Borgs scale of perceived exertion during the field-test was 75.5 (16.4) % of the rating during the peak test (Table 4). The correlation between these ratings was poor (r = -0.179).

### Adverse events

One participant with epilepsy as comorbidity, experienced a mild seizure within 1 hour after the V̇O_2_peak-test. The incident happened at the hospital where the test had been performed. He was admitted to the neurological ward and was observed for 2 hours before he could leave the hospital. The responsible medical doctor considered that it was safe for him to continue his participation in the study. No other adverse events were registered within the course of this study.

## Discussion

This is the first study to assess test-retest reliability of a portable metabolic ergospirometry measurement system during functional activities in people with chronic stroke. The main finding was that the MetaMax II system was reliable for measuring energy expenditure during functional activities in people with mild to moderate stroke.

Our secondary hypotheses were also confirmed as the correlation between V̇O_2_ from the peak-and the field-test reached r>0.6 and because participants spent more than 50% of their peak aerobic capacity during the field-test.

Nunnally and Bernstein (1994) [25], argued that an ICC of ≥ .70 or greater is considered acceptable but higher values are more favorable. In the present study, we found very good reliability (ICC ≥ .80) for walking distance, V̇O_2_, CO_2_, HR and RPE during 6MWT. However, only distance and V̇O_2_ reached an ICC ≥ .90. This is in contrast to the results from Stookey et al. (2013) who reported ICC values ≥ .90 for V̇O_2_, VE and CO_2_ using a portable ergospirometry system (Vmax, Sensor Medics) during the 6MWT in people with stroke. [8] Our ICC values during the field-test were slightly lower compared to the 6MWT. However, all variables except the RER achieved an ICC ≥80. Possible explanations for the slightly lower ICC values during the field-test might be the more complex nature of functional activities, challenging the metabolic demands of our sample more than over-ground walking. Although the limits of agreement depicted for steady state VO_2_ in the present study were a bit larger than those reported by Stookey et al., [8] we believe that our findings indicates that small changes also can be detected by the portable metabolic ergospirometry measurement system during functional tasks.

Interestingly, the relationship between the V̇O_2_ and V̇O_2_peak was almost the same during the 6MWT and the field-test, indicating that performing daily activities at comfortable speed is as strenuous as walking 6 minutes at fast speed in this population. Spruit et al. (2011) found that patients with chronic heart failure spent more than 50% of their V̇O_2_peak while getting dressed measured with portable ergospirometry [12] whereas in our study, we found that people with stroke spent more than 60% of their V̇O_2_peak during functional activities in the standardized field-test.

According to a recent systematic review, it is well documented that stroke survivors consume more energy during walking compared to healthy controls. [7] In addition, Slawinski et al. (2014) assessed the energy cost of stroke participants clearing and skirting obstacles and found that the stroke group had a higher energy cost compared to a matched healthy group. [9] This is in line with the study of Kafri et al. (2014) who reported that stroke survivors have a high metabolic cost during various functional activities. [11] An interesting conclusion from the above mentioned studies is the lack of difference in V̇O_2_ during the activities performed between participants with stroke and the healthy control groups. This is explained by the higher speed of walking in healthy individuals compared to the persons with stroke. A limitation from this research is that the participants’ V̇O_2_peak is estimated indirectly.

So far, no studies have assessed the correlation between V̇O_2_ measured during a treadmill peak-test and a standardized field-test consisting of regular daily activities. According to de Vet et al. (2011), criterion validity is defined as the degree to which the scores of an instrument are an adequate reflection of a gold standard. [26] Furthermore, criterion validity can be subdivided into concurrent validity and predictive validity. Unfortunately, there exist no gold standard for measuring oxygen consumption during a field test. Hence, the criterion validity can only be supported by assessing the strength of the relationship against another clinical measure, which in our study was the peak-test. The relationship between V̇O_2_ in the peak- and field-test was expected to be stable. Hence, the correlation (r>0.6) found in the present study strengthens the validity of the MetaMax II portable ergospirometry system applied during the field test.

### Methodological considerations

Even though a sample size of 31 participants as included in the present study is regarded as a fair sample size according to the COSMIN checklist [26], it is a relatively large sample size compared to similar studies in the stroke population assessing V̇O_2_ measures. [10, 14] Additionally, robust testing of V̇O_2_peak prior to assessing V̇O_2_ during functional activities allowed for calculations of relative strain during the standardized field-test, which may be considered as a strength of the present study.

Our participants were relatively young, however at approximately the same age as similar studies conducted in this population. [7] The participants were also at the higher end of the scale in terms of physical functioning. Hence, the results from the present study should not be generalized to the stroke population at the lower end of the scale in terms of physical functioning. [7]

Our results were averaged over the last 30 seconds of the field-test lasting for more than 4 minutes. For those participants spending longer time to complete the test, the V̇O_2_ during some activities would be higher and exact knowledge about the V̇O_2_ in each activity would have been useful, but this was not the purpose in the present study.

Finally, by using the same testers during both occasions of field-testing may have introduced a potential bias. To minimize such bias, the testers were instructed not to review cardiopulmonary test values from the first test before conducting the second test. In addition, participants were unaware of their test results.

### Clinical implications

Our results suggest that the portable MetaMax II ergospirometry measurement system is a reliable and valid tool for measuring energy consumption during daily activities after stroke. This method has the potential to reliably evaluate cardiorespiratory changes after rehabilitation programs and interventions in the stroke population. Caution should be taken in generalizing the findings from the present study to more severely affected stroke survivors and older individuals.

## Conclusions

This is the first study to establish the test-retest reliability of portable oxygen uptake measurement in people with stroke performing functional activities. Our results demonstrate good to very good test-retest reliability of portable ergospirometry, indicating that the equipment for the most important cardiopulmonary performance parameters could be used outside laboratory settings. The participants in the present study spent a great proportion of their peak aerobic capacity during activities frequently encountered in daily life. Finally, the correlation between V̇O_2_ during the peak incremental treadmill test and the field-test was good, supporting the validity of this method.
